# Chemical Composition and Antibacterial and Antioxidant Activities of Stem Bark Essential Oil and Extracts of *Solanecio gigas*

**DOI:** 10.1155/2022/4900917

**Published:** 2022-07-09

**Authors:** Mequanint Molla Yitayeh, Amanu Monie Wassihun

**Affiliations:** ^1^Department of Organic Chemistry, College of Natural and Computational Science, Debre Tabor University, Debre Tabor, Ethiopia; ^2^Department of Biochemistry, College of Health Science, Debre Tabor University, Debre Tabor, Ethiopia

## Abstract

Herbal medication developed from natural resources has to have antibacterial and antioxidant effects. The aim of this research is to look at the chemical makeup of *Solanecio gigas* (*S*. *gigas*) stem bark essential oil (EO), as well as the effectiveness of EO and extracts (chloroform, ethyl acetate, and methanol) against human pathogenic bacteria and their antioxidant activity. The GC-MS analysis identified 23 components, accounting for 98.7% of the total oil containing Methylene chloride (49.2%), sabinene (10.5%), 1-nonene (11.3%), Terpinen-4-ol (6.9%), Camphene (4.3%), *γ*-terpinene (3.6%), *α*-phellandrene (2.9%) *β*-myrcene (2.6%), 1,2,5-Oxadiazol-3-carboxamide, 4,4′-azobis-2,2′-dioxide (2.4%), *α*-terpinene (1.9%), 1-Octanamine, N-methyl- (1.9%), *ρ*-cymene (1.6%) as major components. The antibacterial efficacy of the EO and extracts (25, 50, 100, and 200 mg/ml) was demonstrated by the inhibitory zones (8.5 ± 0.47–23.3 ± 0.36 and 7.2 ± 0.25–22.0 ± 0.45 mm), respectively. The MIC values of the extracts and the EO were 120–150 and 240 to <1100 *μ*g/ml, respectively. The EO also demonstrated a significant antibacterial impact. The EO and methanolic extract had free radical scavenging activities with IC_50_ value, 13.8 ± 0.48 and 4.2 ± 0.04 *μ*g/ml, respectively. In comparison to the other extracts, the methanolic extract had the greatest phenolics (100.2 ± 0.13 *μ*g GAE/mg of dry extract) and flavonoid contents (112.1 ± 0.18 *μ*g CE/mg of dry extract).

## 1. Introduction

The search for natural-source compounds with pharmacological properties has resulted in the discovery of one of the many new molecules with critical applications [1, 2]. Ethiopia stands out as a potential source of these natural chemicals because it has the highest plant biodiversity in the world and the bulk of its species are yet to be found in terms of their therapeutic potential [3]. Screening EOs and extracts of medicinal plants are popular and scientifically interesting all around the world [1, 3]. The major volatile constituents of EOs have been used in the medicinal, food, and perfume industries in the past because of their antibacterial, gastronomic, and fragrance properties [4].

Also, natural polyphenol compounds are in high demand because of their potential to treat a number of disorders, including diabetes, cardiovascular problems, anti-cancer, anti-inflammatory, and antibacterial properties. In addition, they have potential applications in the food and pharmaceutical industries [5, 6].

Antioxidants have long been used as food additives to prevent food degradation. There are also compounds that react with free radicals, neutralizing them and avoiding or reducing their harmful effects in the human body [7]. Lipid oxidation is to blame for the breakdown of fats and oils, which results in a change in color, flavor, and nutritional value, while oxidative stress plays a role in the etiology of a variety of disorders [8].

Alternative antibiotics, such as plant-based drugs, are being investigated as a possible replacement for traditional antibiotics. As a result, substantial research has been conducted to assess the antimicrobial impact of EOs and extracts, which have demonstrated the potential to suppress the growth of a variety of harmful microorganisms [9].

One of Ethiopia's most important medicinal plants is *S*. *gigas* (Vatke) C. Jeffrey (Asteraceae) is a large rosette plant or shrub with soft woody stems that grows up to 4 meters tall [10]. *S*. *gigas* is only found in Ethiopia, where it goes by the names “Yeshkoko-Gomen,” “Abezenta,” and “Nobe.” It is also one of the most widely used plants in Ethiopian traditional medicine. The aboveground whole plant is used to cure colic, diarrhea, gout, otitis media, typhus, wound dressing, anti-abortifacient, and mental faculty improvement (stems, leaves, and flowers). Extracts from the roots are also used to treat typhoid illnesses [10].

*S*. *gigas* belongs to the Senecioneae family of plants, which includes species including *Cacalia*, *Crassocephalum*, *Emilia*, and *Senecio*, all of which can biosynthesis hepatotoxic pyrrolizidine alkaloids. The plant's widespread use as natural medicine, as well as its taxonomic similarities to the well-studied Senecio species, sparked our interest in conducting scientific research on it [11]. There were few scientific studies on this plant. Here, we report the composition and antibacterial and antioxidant activity of the EO and various extracts of *S*. *gigas* stem bark.

## 2. Materials and Methods

### 2.1. Plant Material

During the flowering season of *S*. *gigas* in Mekane Eyesus in 2021, the stem bark of the plant was taken from the Amhara (South Gondar) region, Ethiopia. Mr. Endale Adamu (botanist, department of biology) confirmed the specimens. The samples were then dried in the shade in the open air until it reached a consistent weight. A laboratory mill was used to grind the dried plant to a fine powder approximately 0.2–0.4 mm. The powder's weight was calculated and stored at 4°C for further investigation.

### 2.2. Extraction Procedure

Fifty grams of the powder was extracted by maceration for 7 days at room temperature with absolute methanol, ethyl acetate, and chloroform (160 mL × 3 for each) [12]. The extracts were then filtered using Whatman filter paper No. 1, and the filtrate was evaporated on a rotary vacuum evaporator to produce solid or semisolid methanol, ethyl acetate, and chloroform extracts, which were kept at 4°C until used.

### 2.3. Isolation of EO

Dried stem barks of *S*. *gigas* (1 kg) were submitted to hydro-distillation in a Clevenger-type apparatus [13]. The isolation experiment was carried out continuously on a heating mantle at a temperature of 60–80°C for 4 h or until no further oil was extracted. The distilled oil was extracted with diethyl ether and dried over anhydrous sodium sulfate. After filtration, the sample is stored in a dark bottle at 4°C until tested and analyzed. The yield of the obtained EO was about 1.2 g (0.12%) based on the dry weight of the plant material.

### 2.4. GC-MS Analysis

A Shimadzu GC-MS (GC-17A) equipped with a ZB-1 MS fused silica capillary column (30 m × 0.25 mm i.d., film thickness of 0.25 *μ*m) was used to analyze the EO [14]. An electron ionization device with ionization energy of 70 eV was employed for GC-MS detection. At a steady flow rate of 1 mL·min^−1^, helium was used as the carrier gas. Temperatures for the injector and MS transfer line were set at 220 and 290 degrees Celsius, respectively. The oven temperature was designed to rise from 50 to 150°C at a rate of 3°C min^−1^, then to stay at that temperature for 10 minutes before being raised to 250°C at a rate of 10°C min^−1^. In the split-less mode, diluted samples (1/100, v/v, in methanol) of 1.0 *μ*L were manually injected. Peak area normalization was used to express the relative percentages of the oil elements as percentages. The EO molecules were identified using GC retention time on a ZB-1 capillary column, computer mass spectral matching with those in the Wiley 6.0 GC/MS library, and literature data [15].

### 2.5. Determination of Phytoconstituent

#### 2.5.1. Determination of Total Phenolic Content

The total phenolic content (TPC) in extracts was determined using the Folin-Ciocalteu method [16] with slight modifications. In a nutshell, 100 *μ*L of extract (1 mg/mL) were diluted to 4.6 mL before being mixed with 100 *μ*L of Folin-Ciocalteu reagent. The mixture was then allowed to sit for 3 min before being added to 300 *μ*L of 2% Na_2_CO_3_. After 90 minutes of incubation at 25°C, the absorbance was measured at 750 nm. The results were expressed as a ratio of *μ*g GAE/mg dry extract.

#### 2.5.2. Determination of Total Flavonoid Content

The total flavonoid content (TFC) of extracts was measured using previously published techniques [16]. After 5 min at room temperature, an aliquot of 500 *μ*L of each *S*. *gigas* leaf extract (1 mg/mL) was added to 75 *μ*L of sodium nitrite solution (5%) mixed with 150 *μ*L of aluminum chloride (10%), 500 *μ*L of NaOH reagent (1 M), and the absorbance was measured at 510 nm. The catechin equivalent (CE) (*μ*g CE/mg dry extract) was used to calculate TFC.

### 2.6. Determination of Biological Activities

#### 2.6.1. Antibacterial Bioassays

The bioactive compounds' antibacterial activity was assessed using a modified agar disk diffusion method [17]. The antibacterial activity of the samples was tested using bacteria from the American Type Culture Collection (ATCC), which included gram-positive bacteria like *S*. *aureus* (ATCC 31488) and *S*. *pyogenes* (ATCC 27853), as well as gram-negative bacteria like *E*. *coli* (ATCC 25922) and K. pneumonia (ATCC 25922). Each test microbe was seeded in Mueller Hinton broth, homogenized, and then swamped in a sterilized Petri dish to obtain a uniform depth. Antibacterial experiments were conducted with four different concentrations (serial dilutions of 200, 100, 50, 25, and 12.5 mg/mL) produced in 5% dimethyl sulfoxide (DMSO). Sterile filter paper disks (Whatman No. 1, diameter = 6 mm) were soaked in the plant extracts and EO, and placed it on Mueller-Hinton agar plates. Plates were incubated at 35 ± 2°C for 24 hours. A positive control of gentamicin (10 mg/disc) was placed on the center of each petri-dish, whereas a negative control of 5% DMSO was employed. The antibacterial zone of inhibition was estimated by millimeters (mm).

#### 2.6.2. Determination of Minimum Inhibitory Concentration

The lowest sample concentration that prevented observable growth was determined as the minimum inhibitory concentration (MIC). To establish the minimal inhibitory concentrations, the EOs and extracts were serially diluted from 25 to 225 *μ*g/ml. About 50 *μ*l sterile Mueller Hinton Broth (MHB) was added into 96-well plate [18]. Separate wells were used for the sterility and growth controls, with the sterility control containing only Oxoid® MHB and the growth control containing both MHB and the test organism. After adding 50 *μ*l of bacterial suspension (105 CFU/mL) to each row, the microplate was covered and incubated at 37°C with 100% relative humidity overnight (except for the sterility control). The following morning, each well-received 50 *μ*l of a 0.2 mg/ml p-iodonitrotetrazolium violet (INT, Sigma-Aldrich) solution. Growth inhibition was indicated by a clear solution or a marked decrease in color response. The EO and extracts were dissolved in a 5% dimethyl sulfoxide (DMSO) solution to determine MICs of this plant sample.

#### 2.6.3. Free Radical Scavenging Activity

Using the method described earlier, the antioxidant activity of the EO and methanol extract was determined by their ability to scavenge 2,2-diphenyl-1-picrylhydrazyl (DPPH) stable radicals [19]. 3 mL of methanolic extract and 1 mL of freshly prepared 0.1 mM DPPH methanolic solution were combined thoroughly and kept in the dark for 60 minutes. A spectrophotometer was used to measure the absorbance of the reaction mixture at 517 nm. The blank was made by substituting methanol for the extract (1 mL). The percentage of free radical scavenging activity was calculated as follows:

  The % antioxidant activity calculated as (*I*%) = [(Abs control − Abs sample)]/(Abs control)] × 100.
  Abs control = absorbance of DPPH radical + methanol
  Abs sample = absorbance of DPPH radical + sample extract

All of the tests and analyses were performed three times. From the graph plotting inhibition percentage against extract concentrations, the extract concentration providing 50% inhibition (IC_50_) was derived. As a positive control, an ascorbic acid methanol solution was employed.

### 2.7. Statistical Analysis

All tests were carried out in triplicate, and data was expressed as mean standard deviation (SD) or standard error of mean (SEM), excel, and chemdraw (to draw different chemical structure). The results were thought to be statistically significant.

## 3. Results

The extracts of *S*. *gigas* stem bark yielded different percent based on room temperature dry weight by different solvents. The percentage quantities of the extracts with methanol, ethyl acetate and chloroform solvents were presented in Table 1.

### 3.1. Qualitative Phytochemical Analysis

In qualitative analysis of stem bark methanolic extract of *S*. *gigas* exhibited positive results from ten phytochemical tests. Nine phytochemical tests were positive in the extract of the plant. The stem bark methanolic extract of the plant showed the presence of alkaloids, flavonoids, steroids, tannins, saponins, anthraquinones, terpenoids, glycosids, and phenolic compounds, and absence of reducing sugar (Table 2). These phytochemical components are known to support bioactive activities in medicinal plants and are hence responsible for the antioxidant and antibacterial properties.

### 3.2. Chemical Composition of the EO

**Figure 1:**
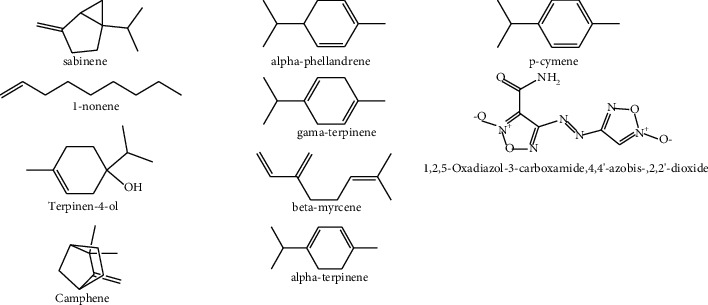
The major constituents of EO of *S*. *gigas* stem bark.

Yellowish oil was obtained by hydro-distilling the stem bark of *S*. *gigas* stem bark. The oil was analyzed using GC-MS, and twenty-three distinct chemicals were discovered, accounting for 97.2% of the total oil.  Table 3 lists the detected chemicals in the order in which they eluted on a ZB-1 capillary column. The oil contains a complex blend of monoterpene hydrocarbons, oxygen-containing mono-terpenes, and other important phytochemicals. As shown in Table 3, the major compounds detected in the oil were Methylene chloride (49.2%), sabinene (10.5%), 1-nonene (11.3%), Terpinen-4-ol (6.9%), Camphene (4.3%), *α*-phellandrene (2.9%), *γ*-terpinene (3.6%), *β*-myrcene (2.6%), 1,2,5-Oxadiazol-3-carboxamide, 4,4′-azobis-, 2,2′-dioxide (2.4%), *α*-terpinene (1.9%), 1-Octanamine, N-methyl-(1.9%), *ρ*-cymene (1.6%) (Figure 1), and along with major constituents, minor constituents were also reported.

### 3.3. Total Phenols and Flavonoids

Antioxidant, antibacterial, and other biological properties have been found for phenolic compounds. Folin-Ciocalteu technique was used to evaluate total phenolic components in extracts, which were expressed as Gallic acid equivalents (GAEs).

Table 4 reveals that the phenolic compound content in methanolic extract was highest (100.2 ± 0.13 *μ*g GAE/mg), followed by ethyl acetate extract (57 ± 0.02 *μ*g GAE/mg). The chloroform extract contains the smallest phenolic chemicals (27.4 ± 0.53 *μ*g CE/mg). Total flavonoids in catechin equivalent were calculated (CE). The maximum concentration of flavonoids was found in *S*. *gigas* methanol extract (112.1 ± 0.18 *μ*g CE/mg), followed by Ethyl acetate extract (67.2 ± 0.24 *μ*g CE/mg) and chloroform extract (40.3 ± 0.23 *μ*g CE/mg).

### 3.4. Determination of Biological Activities

#### 3.4.1. Antibacterial Activity

The presence of inhibitory zones was used to qualitatively and quantitatively analyze the antibacterial activity of *S*. *gigas* stem bark EO and extracts against the microorganisms used. As shown in Table 5, the EO at 200 mg/ml exhibited potent inhibitory effect against the tested bacterial pathogens, *E*. *coli* (ATCC 25922) was found to be highest inhibited bacterial pathogen by the EO with their respective diameter zones of inhibition of 23.3 ± 0.36 mm, and the rest of the bacterial strains, *K*. *pneumonia* (ATCC 27853), *S*. *aureus* (ATCC 31488) and *S*. *pyogenes* (ATCC 27853) were inhibited in a good way, with diameter of zones of inhibition ranging from 17.2 ± 0.40–19.3 ± 0.52 mm by this EO of the plant.

**Figure 2:**
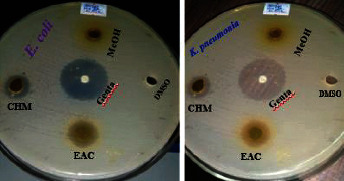
Zone of inhibition of *E*. *coli* and *K*. *pneumonia* by extracts at 200 mg/ml.

In addition, the methanolic and ethyl acetate extract shown antibacterial action against the bacteria tested. Also, at 200 mg/ml, the diameters of methanolic and ethyl acetate extract zones of inhibition against the studied microorganisms were 17.5 ± 0.50–22.0 ± 0.45 mm and 17.9 ± 0.17–21.9 ± 0.51 mm (Figure 2), respectively at maximum concentration. However, chloroform extract exhibited moderate effect of antibacterial activity against tested bacterial pathogens with diameters of zones of inhibition ranging from 13.2 ± 0.39–16.4 ± 0.30 mm at 200 mg/ml. Methanolic extract exhibited potent inhibitory effect of antibacterial activity against the tested bacteria as compared to ethyl acetate and chloroform extracts. In this study, the oil and extracts exhibited higher antibacterial activity in regard to gram-negative bacteria than gram-positive bacteria.

#### 3.4.2. Minimum Inhibitory Concentrations

As shown in Table 6 the EO's MIC values against the tested bacterial strains were lower than the extracts. With MIC values of 120 and 150 *μ*g/ml, the EO had a great antibacterial action against two Gram-negative bacteria and two Gram-positive bacteria, respectively. In contrast to chloroform extract, ethyl acetate and methanol extracts had a significant antibacterial impact. Methanol, ethyl acetate, and chloroform extract all had better antibacterial effects against all of the tested bacterial strains with MICs ranging from 240 to 1100 *μ*g/ml, but chloroform extract had a mild to moderate antibacterial effect against all Gram-negative and Gram-positive bacteria with MIC values ranging from 1000 to 1100 *μ*g/ml, respectively.

#### 3.4.3. Antioxidant Activity

**Figure 3:**
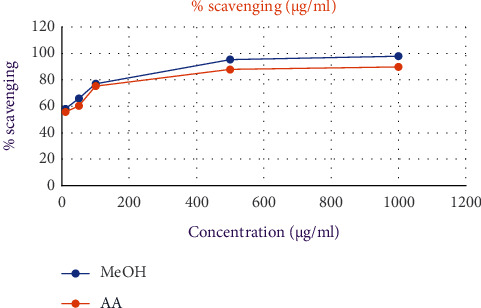
Free radical scavenging activity of methanolic extract from *S*. *gigas* stem bark and standard (ascorbic acid).

**Figure 4:**
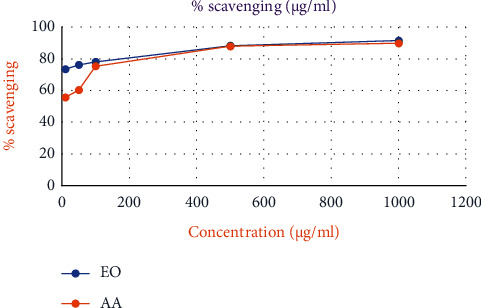
Free radical scavenging activity of EO from *S*. *gigas* stem bark and standard (ascorbic acid).

The DPPH test was performed to evaluate the free radical scavenging activities of the methanolic extract and EO, and the reaction followed a concentration-dependent pattern (Figures 3 and 4).  Table 7 shows that higher *S*. *gigas* concentrations result in a larger inhibition ratio (percentage). The ascorbic acid standard (AA = 4.7 ± 0.02 *μ*g/ml) was used to compare the IC_50_ values of EO and methanolic extract. The best antioxidant activity is shown by a reduced IC_50_ value. Methanolic extract and EO both had IC_50_ values of 4.2 ± 0.04 and 13.8 ± 0.48 g/ml, respectively. Methanolic extract was found to be superior to both EO and the standard (ascorbic acid) in terms of free radical scavenging.

## 4. Discussion

Phytochemicals such as alkaloids, flavonoids, steroids, tannins, saponins, anthraquinones, terpenoids, glycosids, and phenolic compounds were discovered in plant methanol extract. These are recognized to have both therapeutic and bioactive characteristics. Several researchers have reported the analgesic [20], antispasmodic and antibacterial [21, 22] properties of alkaloids. Glycosides are known to lower the blood pressure according to many reports [23]. Apoptosis, anti-aging, anti-carcinogen, anti-inflammation, anti-atherosclerosis, cardiovascular protection, and improvement of endothelial function are some of the biological features of phenolic compounds, as well as suppression of angiogenesis and cell proliferation activities [24]. Saponins, which are known to have an anti-inflammatory impact, were also discovered in the plant extracts [25]. Antibacterial properties have been reported for steroids [26] and they are crucial molecules, especially because of their interactions with other substances like sex hormones [27]. Tannins inhibit the growth of many fungi, yeasts, bacteria, and viruses [28]. Analgesic and anti-inflammatory properties are attributed to terpenoids and tannins [29].

Plant based secondary metabolites such as EO and extracts are widely used in the food industry and considered Generally Recognized as Safe (GRAS). Various publications have documented the antimicrobial activity of the EOs and plant extracts [30]. Also, several researchers reported mono- and sesquiterpenoids as the major components of EOs, which are phenolic in nature. It seems reasonable to assume that their antimicrobial mode of action might be related to the phenolic compounds present [31, 32]. Most of the studies on the mechanism of phenolic compounds have focused on their effects on cellular membranes. Phenolic compounds not only attack cell walls and cell membranes, thereby affecting their permeability and release of intracellular constituents (e.g. ribose, Na glutamate) but they also interfere with membrane functions (electron transport, nutrient uptake, protein, nucleic acid synthesis and enzyme activity) [33]. Thus, active phenolic compounds might have several invasive targets which could lead to the inhibition of bacterial pathogens [33, 34]. There was a study that found the ginger leaves and chia seeds with higher phenolic content showed more significant potential to eliminate the pathogenic bacteria [35, 36]. Plant phenolic content has been associated to antioxidant activity in several studies, most likely due to their redox properties, which allow them to serve as reducing agents, hydrogen donors, and singlet oxygen quenchers [37].

Also, the results of the antibacterial screening showed that the stem bark EO and extracts of methanol, ethyl acetate and chloroform have potential antibacterial effect against Gram-positive bacteria such as *S*. *aureus* (ATCC 31488) and *S*. *pyogenes* (ATCC 27853), as well as Gram-negative bacteria such as *E*. *coli* (ATCC 25922) and *K*. *pneumonia* (ATCC 27853). This might be the result of the major components such as Methylene chloride (49.2%), sabinene (10.5%), 1-nonene (11.3%), Terpinen-4-ol (6.9%), Camphene (4.3%), *α*-phellandrene (2.9%), *γ*-terpinene (3.6%), *β*-myrcene (2.6%), 1,2,5-Oxadiazol-3-carboxamide, 4,4′-azobis-, 2,2′-dioxide (2.4%), *α*-terpinene (1.9%), 1-Octanamine, N-methyl- (1.9%), *ρ*-cymene (1.6%) present in the stem bark EO of *S*. *gigas* (Figure 1) and these findings are in agreement with previous reports [38].

EO, which are odorous and volatile products of plant secondary metabolism, have wide applications in the food flavoring and preservation industries [39]. In addition, it is also possible that the minor components might be involved in some type of antibacterial synergism with other active components of EO, as evident by the previous work [40]. The results from viable count assay revealed that exposure of the MIC concentration of the stem bark essential oil and extracts had a severe effect on the cell viability of the tested bacteria. All the strains of *S*. *aureus* (ATCC 31488) and *S*. *pyogenes* (ATCC 27853), *E*. *coli* (ATCC 25922) and *K*. *pneumonia* (ATCC 27853) were found sensitive to the essential oil and the extracts. The investigated plant EO showed maximum antibacterial activity against Gram-negative, *E*. *coli*, and *K*. *pneumonia* [41] than Gram-positive, *S*. *aureus* and *S*. *pyogenes*. The present results agree with that of [42] who reported that the EO isolated from the freshly collected *S*. *gigas* flower can inhibit different bacterial and fungal species.

It's possible that the difference in sensitivity between Gram-negative and Gram-positive bacteria is due to differences in cell wall structure. The cell wall of Gram-positive bacteria is made up of 70–100 layers of peptidoglycans [43]. Peptidoglycan is made up of N-acetylglucosamine and N-acetylmuramic acid, which are connected together by peptide side chains and cross bridges [44, 45]. As an explanation, this is unquestionably an oversimplification, and additional mechanisms are almost surely at work. The production of the lactamase enzyme in the periplasmic gap between the thin outer membrane and the cytoplasmic membrane causes Gram-negative bacteria to become resistant to antibiotics like penicillin [14].

Furthermore, the DPPH radical is a free radical that has been widely used as a tool to estimate the free radical scavenging activity of antioxidants. When antioxidants interact with DPPH, they either transfer electrons or hydrogen atoms to it, neutralizing its free-radical nature [19, 46]. In the present study, EO and methanolic extract showed higher or lower antioxidant activities as compared to the standard ascorbic acid (Figures 3 and 4). This is due to most bioactive compounds such as polyphenols, including tannins and flavonoids, existing in higher polar extracts. Polyphenols are one of the major plant compounds with antioxidant activity. The antioxidant activity of phenolic compounds is reported to be mainly due to their redox properties [47], which can play an important role in absorbing and neutralizing free radicals, quenching singlet and triplet oxygen, or decomposing peroxides. Furthermore, phenolics were discovered to be one of the most abundant ingredient groups in a methanol extract of *S*. *gigas* stem bark. This is because methanol extract has a higher concentration of bioactive chemicals than other organic extracts. Several studies have stressed the importance of phenolic compounds in scavenging free radicals [47].

Organic extracts may be more beneficial than isolated constituents because other compounds present in the extracts can change the chemical or biological properties of bioactive individual components [48, 49]. In this study, phenolics were found to be one of the constituents in methanolic extract. This is due to the presence of more bioactive compounds in methanolic extract as compared to EO. The key role of phenolic compounds in scavenging free radicals has been emphasized in several reports [50, 51].

## 5. Conclusion

Alkaloids, flavonoids, steroids, tannins, saponins, anthraquinones, terpenoids, glycosids, and phenolic compounds were found in a methanolic extract of *S*. *gigas* stem bark. According to the researchers, these molecules could be utilized as preservatives to prevent food-borne diseases as well as natural antioxidants to reduce oxidative stress in humans. The results of this study suggest that stem bark EO and extracts could be used in the food industry. The natural origins of the EO and extracts, which consumers find comforting and are good for the environment, as well as the extremely low risk of pathogens developing resistance to the mixture of components that make up the oil and extracts, which appear to have a wide range of antibacterial mechanisms, are the main reasons for their suitability. As a result of these beneficial properties, food safety and shelf life may be increased. This applies to medications as well as the food and cosmetics industries. As a result of the aforesaid findings, it may be inferred that natural antibacterial and antioxidant agents generated from *S*. *gigas* stem barks will be suitable for food industry applications, as plant-derived natural antimicrobials have been used by mankind for ages with little or no toxicity. The antioxidant and antibacterial activities of the extracts were impacted by the relative amounts of TPC and TFC, as well as the EO, due to the presence of major components. As a result, further rigorous study is needed to isolate the beneficial component, conduct toxicity testing, and conduct clinical trials.

## Figures and Tables

**Table 1:** Different mass of extracts were recorded from the stem barks of *S*. *gigas*.

Solvent	Amount mass taken (gm)	Stem bark	
		Extracted amount (gm)	Percentage yield
Chloroform	50	2.5	5
Ethyl acetate	50	3.75	7.5
Methanol	50	6.2	12.4

**Table 2:** Phytochemical analysis of *S*. *gigas* stem bark methanolic extract.

Compounds	Formation	Stem bark
Steroids	The development of greenish coloration indicates the presence of steroids	+
Flavonoids	Formation of intense yellow color, which becomes colorless on the addition of dilute acid, indicates the presence of flavonoids	++
Saponins	Formation of foam indicates the presence of saponins	++
Tannins	The black color indicates the presences tannins	+++
Anthraquinone	The violet color shows the presence of anthraquinone	+
Sugars	—	−
Terpenoids	Grayish color formation indicates the presence of terpenoids	++
Phenolic compounds	Phenolic compound was confirmed by the development of a greenish black color	+++
Alkaloids	Formation of pale-yellow precipitate indicates the presence of alkaloids in the sample	+++
Glycoside	A reddish brown colour indicated the presence of the steroidal ring, i.e., the glycone portion of the glycoside	+

Key: +++ indicates the presence of very intensive compounds, ++ indicates the presence of intensive compounds, + indicates the presence of less intensive compounds, − absent.

**Table 3:** The constituents of the EO of *S*. *gigas* stem barks.

PK	RT (min)	Content in oil (%)	Constituent
1	7.0198	49.2418	Methylene chloride
2	8.2985	1.2784	Cyclopropane carboxamide
3	9.8535	0.4463	o-Veratramide
4	9.9773	0.5861	Ethyl chloride
5	11.4652	4.3124	Camphene
6	12.9133	11.2511	1-Nonene
7	12.9262	10.5123	Sabinene
8	14.473	0.4266	(-)-Norephedrine
9	14.5233	0.6594	Amphetamine
10	15.2923	1.8756	*α*-terpinene
11	15.8734	1.5543	*ρ*-cymene
12	16.163	1.1237	2-Octanamine
13	16.2575	2.6376	*β*-myrcene
14	16.386	1.2616	2,5-Dimethoxy-4-(methylsulfonyl)amphetamine
15	16.9603	2.8753	*α*-phellandrene
16	17.6248	1.1432	1-Octanamine, N-methyl-
17	18.3755	3.6233	*γ*-terpinene
18	18.9823	2.4324	1,2,5-Oxadiazol-3-carboxamide, 4,4′-azobis-, 2,2′-dioxide
19	21.9446	0.9445	Linalool
20	22.0036	0.8989	1-Octadecanamine, N-methyl-
21	22.129	1.2077	Amphetamine
22	22.2462	1.8767	1-Octanamine, N-methyl-
23	25.3195	6.9344	Terpinen-4-ol
Total		97.2414	

**Table 4:** Showing total phenol and flavonoid content of different extracts of *S*. *gigas* stem barks.

Test sample extract	TPC (*μ*g GAE/mg of dry extract)	TFC (*μ*g CE/mg of dry extract)
Chloroform	27.4 ± 0.53	40.3 ± 0.23
Ethyl acetate	57 ± 0.02	67.2 ± 0.24
Methanol	100.2 ± 0.13	112.1 ± 0.18

Data is represented as mean ± SD of three triplicate experiments.

**Table 5:** Zone of inhibition (mm) of antibacterial activity of the investigated plant in agar diffusion test.

Extracts	Conc. mg/ml	Bacterial strains			
		*E*. *coli*	*K*. *pneumonia*	*S*. *aureus*	*S*. *pyogenes*
EO	200	23.3 ± 0.36	19.3 ± 0.52	17.2 ± 0.40	18.1 ± 0.40
	100	15.8 ± 0.72	13.9 ± 0.60	13.9 ± 0.85	12.7 ± 0.64
	50	14.9 ± 0.06	13.4 ± 0.35	10.4 ± 0.40	9.5 ± 0.40
	25	10.8 ± 0.76	10.2 ± 0.72	9.1 ± 0.32	8.5 ± 0.47

MeOH	200	20.4 ± 0.45	22.0 ± 0.45	17.5 ± 0.50	18.4 ± 0.51
	100	16.4 ± 0.32	17.1 ± 0.61	15.1 ± 0.46	16.0 ± 0.65
	50	12.1 ± 0.51	10.1 ± 0.15	10.4 ± 0.32	9.3 ± 0.26
	25	9.9 ± 0.12	9.4 ± 0.38	8.4 ± 0.36	7.4 ± 0.46

EAC	200	21.9 ± 0.51	18.1 ± 0.40	17.9 ± 0.17	19.2 ± 0.40
	100	16.9 ± 0.36	14.1 ± 0.31	14.6 ± 0.40	16.8 ± 0.20
	50	13.2 ± 0.15	12.2 ± 0.15	10.4 ± 0.51	8.6 ± 0.36
	25	10.8 ± 0.40	10.1 ± 0.23	9.4 ± 0.36	7.2 ± 0.25

CHM	200	16.4 ± 0.30	14.0 ± 0.60	14.3 ± 0.41	13.2 ± 0.39
	100	12.2 ± 0.31	11.2 ± 0.75	11.4 ± 0.52	9.4 ± 0.29
	50	10.1 ± 0.23	9.1 ± 0.29	9.3 ± 0.32	7.8 ± 0.28
	25	8.2 ± 0.24	7.4 ± 0.1	7.4 ± 0.31	7.5 ± 0.07

Genta	10 mg/disc	24.7 ± 0.3	24.0 ± 0.25	22.5 ± 0.70	22.2 ± 0.21

EO: essential oil; DMSO, the negative control, has no effect.

**Table 6:** Minimum inhibitory concentration in *μ*g/ml of the plant EO and extracts against bacterial strains.

Bacterial strains	EO and extracts			
	EO	MeOH	CHM	EAC
*K*. *pneumonia*	120	240	1000	400
*E*. *coli*	120	240	1000	400
*S*. *aureus*	150	245	1100	450
*S*. *pyogenes*	150	245	1100	450

**Table 7:** Percentages and IC_50_ values (*μ*g/ml) for radical scavenging activity of EO and extracts (mean ± standard deviation).

Concentration (*μ*g/ml)	EO	AA	MeOH
10	73.4 ± 0.36	55.7 ± 0.15	58.1 ± 0.31
50	76.1 ± 0.15	60.3 ± 0.21	65.9 ± 0.2
100	78.1 ± 0.15	75.3 ± 0.2	77.1 ± 0.31
500	88.2 ± 0.81	87.9 ± 0.1	95.4 ± 0.50
1000	91.5 ± 0.15	89.8 ± 0.2	97.9 ± 0.2
IC_50_ value	13.8 ± 0.48	4.7 ± 0.02	4.2 ± 0.04

## Data Availability

The data used to support the findings of this study are available within the article.
